# Physiological and biochemical responses of *Tanacetum balsamita* L. to the foliar application of Dobogen biostimulant, glucose and KNO_3_ under salinity stress

**DOI:** 10.1038/s41598-022-13150-z

**Published:** 2022-06-04

**Authors:** Mohammad Bagher Hassanpouraghdam, Lamia Vojodi Mehrabani, Mehdi Kheiri, Antonios Chrysargyris, Nikolaos Tzortzakis

**Affiliations:** 1 Department of Horticulture, Faculty of Agriculture, University of Maragheh, Maragheh, 55181-83111 Iran; 2 Department of Agronomy and Plant Breeding, Azarbaijan Shahid Madani University, Tabriz, 5375171379 Iran; 3 Department of Agricultural Sciences, Biotechnology and Food Science, Cyprus University of Technology, 3036 Limassol, Cyprus

**Keywords:** Plant physiology, Plant stress responses

## Abstract

Salinity is one of the predominant abiotic stress factors that influence the growth and productivity of plants. Salinity adversely impacts the growth responses via ionic toxicity, osmotic stress, impaired nutrients uptake, hormonal disparity, and the over-production of reactive oxygen species. To study the effects of salinity stress (0, 50, 100, and 150 mM) and foliar treatments (dH_2_O, 2 g L^−1^ Dobogen biostimulant, 2 g L^−1^ KNO_3,_ and 2 g L^−1^ d-glucose) on the growth and physiological responses of *Tanacetum balsamita,* a factorial experiment was conducted based on the completely randomized design at the research greenhouse of Azarbaijan Shahid Madani University, Iran. The results showed the significant interaction effects of salinity and foliar sprays on chlorophyll a, K^+^, Na^+^, Mg^2+^, Fe^2+^, Zn^2+^, Mn^2+^, and Si content, K/Na ratio, and total phenolics and flavonoids content. The highest phenolic content was acquired with 100 mM salinity and foliar spray of Dobogen and glucose, 50 mM NaCl × KNO_3_ application, and 50 mM salinity × no-foliar application. The highest K/Na ratio was observed in control plants and controls × KNO_3_ and/or Dobogen application. The greatest Si content was recorded with no-salinity × Dobogen and KNO_3_ applications and no-saline × no-foliar (control) plants. The independent effects of treatments influenced malondialdehyde, flavonoids, proline contents, and catalase activity. Chlorophyll b and superoxide dismutase were affected by the salinity. Total soluble solids and Ca^2+^ content were responsive to the foliar applications. Malondialdehyde and proline content was the highest at 150 mM salinity. Salinity adversely affected the physiological responses of costmary. However, foliar treatments partially ameliorated the salinity effect, and the results with more detailed studies would be advisable to the extension section and pioneer farmers.

## Introduction

Costmary (*Tanacetum balsamita* L. from Asteraceae), a traditional medicinal plant of Iranian origin, has been in common endemic use as flavoring, cardiotonic, and flatulence^1^. Costmary is a volatile oil-bearing plant, and its essential oil has been a significant source of flavoring in the food industry. The crop is under production in many parts of Iran and some European countries^1,2^.

Salinity stress is the predominant abiotic stressor that limits plants' growth, development, and productivity by reducing the rhizosphere's osmotic potential, ionic imbalances, oxidative stress, damage to the cell membranes, photosynthesis impedance and a tremendous increase in light-dependent respiration^3–5^. Salinity causes massive chaos in the absorption, translocation, and distribution of essential nutrients and drastically impacts plants' growth and physiological responses. The osmotic and oxidative stress induced by the salinity damage the proteins, DNA, and cell membranes, disrupt chloroplast apparatus structure and photosynthesis potential and ultimately decline productivity and crop quality^1,3–5^. Salinity tolerance is a complicated phenomenon controlled by a cluster of genes mediating several physiological and biochemical processes^6,7^.

The typical way to compensate for soil nutrient shortages is to use chemical fertilizers. However, several surveys demonstrate the deteriorative effects of continuous soil-based chemical fertilizers on ecosystems, with drastic negative impacts such as nitrate and heavy metals accumulation and eutrophication. Foliar application is beneficial in reducing chemical fertilizer inputs and even is more influential in meeting the emergent nutrients need of plants and sometimes meet the long-term micronutrients needs and hence improves the plant growth, development, and productivity^5,8,9^. Potassium is a significant nutrient element with un-substitutable roles in several vital physiological processes, such as stomatal movement, osmotic regulation, enzymatic activity, water balance maintenance, carbohydrate translocation, membrane polarity & stability, and pH equilibrium, as well as plays chief roles in the assimilates translocation^10–13^ and in secondary metabolism of plants^14^. Potassium has a pivotal role in the growth, development, and enhancement of tolerance and survival rate under stressful conditions.

Moreover, potassium has direct and indirect actions in ROS scavenging machinery of plants via the activation of both the enzymatic and non-enzymatic routes. In sunflower plants, K foliar application improved physiological traits like chlorophyll content, soluble proteins content, and CO_2_ acquisition under salinity^15^. Seemingly, potassium manages these crucial actions through accelerated enzymatic functions, leading to improved metabolic activities.

Currently, sugars are commonly used as growth regulators that mediate plant development and gene expression under stressful environments^16,17^. Luo et al.^16^ noted that trehalose, a non-reduced disaccharide, had an antioxidant role, protected proteins, and elicited genes involved in the detoxification process in response to environmental stress factors. Studies on *Ficus carica* showed that the mild salinity (100 mM) enhanced the transcription of genes involved in carbohydrate metabolism and translocation. The enhanced levels of sorbitol and sucrose by the upstream regulation of sorbitol dehydrogenase and sucrose synthase gene decline the adverse-side effects of salinity^18^. In most species sensitive to salt stress, potassium ions' concentration, distribution, relocation, and partitioning is disrupted in the metabolizing organs, especially in leaves, leading to the retarded growth and reduced productivity^15,18^. Under the mentioned conditions, foliar K application tranquilizes salinity depression and partially resumes the normal metabolism and growth^10^.

Salicylic acid (SA) is a predominant signaling compound in plants that triggers a cascade of events in response to the diverse biotic and abiotic stress factors by the activity initiation of a series of stress-responsive genes. Furthermore, SA plays a role in the ions' translocation, especially under stress conditions. The improved K translocation towards the aerial parts secures the plant survival under a saline-sodic environment^2,19,20^. Plants treated with salicylic acid were more tolerant to reactive oxygen species (ROS) deterioration under stressful environments^19^. In *Tanacetum parthenium*, SA application significantly reduced the salinity effects of NaCl and CaCl_2_ and improved the growth and productivity of plants^2^. Therefore, having the protocols reduce salinity's dangerous effects is of particular interest. With the progressed precipitation declines and the coincident salinity incidence in most localities of Iran, the study of foliar applications of promising reagents to overcome salinity depressions is crucially important. Therefore, the present study aimed to evaluate i) salinity effects on costmary and ii) the foliar implementation of Dobogen biostimulant, KNO_3,_ and glucose on the growth and physiological traits of costmary under salinity stress.

## Results and discussions

### Growth-related traits

Root dry weight, petiole length and leaf length, and width were affected by salinity (*P* ≤ 0.01) (Table 1). Leaf length was increased in control and 50 mM NaCl treatments (Table 2). Meanwhile, the top plant height, petiole length, and leaf width were acquired by control plants, while salinity of ≥ 50 mM decreased those parameters (Table 2). Similar results were reported by Valizadeh-Kamran et al.^21^ on *Lavandula stoechas* and by Chrysargyris et al.^22^ in *Mentha spicata* grown under saline conditions. Moreover, a reduction in plant height due to salinity has been reported in *Solanum nigrum*^23^ and *Tanacetum parthenium*^2^. Other studies have shown adverse salinity effects on yield, morphological traits, and plant height. The reasons ascribed to this are reduced photosynthesis, chlorophyll structural breakdown, diminished vital metabolites biosynthesis, and the toxic effects of Na^+^ and Cl^−^, which meaningfully reduce cell turgor, metabolism, and function and eventually impacts plant productivity^3,4,24^. Salinity alters plants' physiological and biological dynamics, and the variations depend on the time and intensity of stress exposure. The primary effect of salinity stress is the over-accumulation of Na^+^ and Cl^−^ ion in the plant tissue, inducing ionic disequilibrium and various physiological disorders. The enhanced Na^+^ uptake prevents K^+^ acquisition, which impedes several physiological processes. ROS radicals are over-expressed with stress endurance, and the macro-molecules like proteins, lipids, and DNA are damaged^3,9,10,12,21^. The results from the present experiment revealed the apparent side-effects of salinity on the mentioned traits (except root dry weight) of plants.

**Table 1:** ANOVA for the effect of salinity (0, 50, 100, and 150 mM NaCl) and foliar applications (no-foliar, KNO_3_, glucose, and Dobogen) on the root and aerial parts dry weight, plant height, leaf length and width, petiole length, the total soluble solids content of *Tanacetum balsamita* plants grown hydroponically in perlite.

Source of variation	df	Biomass DM	Root DM	Plant height	Leaf length	Leaf width	Petiole length	TSS
		Mean square	Mean square	Mean square	Mean square	Mean square	Mean square	Mean square
Salinity (S)	3	85.20^ns^	72.65*	213.40*	28.83*	11.17*	93.40*	0.50^ns^
Foliar (F)	3	30.17^ns^	7.86^ns^	49.55^ns^	5.383^ns^	1.78^ns^	27.54^ns^	1.50*
S × F	9	1263.72^ns^	26.05^ns^	31.52^ns^	0.61^ns^	0.67^ns^	28.31^ns^	0.27^ns^
Error		49.188	21.809	44.587	3.089	0.933	34.06	0.365

df, degree of freedom; ns, non-significant.

*Significant difference at P ≤ 5%, following two-way ANOVA.

**Table 2:** Mean comparisons for the effects of salinity (0, 50, 100, and 150 mM NaCl) on plant height, root DM, leaf length, leaf width, and petiole length of *Tanacetum balsamita* plants grown hydroponically in perlite.

Salinity levels (mM)	Plant height (cm)	Root DM (g)	Leaf length (cm)	Leaf width (cm)	Petiole length (cm)
0	42^a^	21.8^a^	9.1^a^	3.9^a^	7.9^a^
50	37^b^	23.0^b^	8.7^a^	3.3^b^	3.9^b^
100	36^b^	26.4^a^	6.8^b^	3.2^b^	2.8^b^
150	36.2^b^	26.2^a^	6.5^b^	3.0^b^	2.7^b^

Significant differences among salinity treatments are indicated by the different Latin letters according to Duncan’s multiple range test.

### Total soluble solids content

Salinity effects were not significant on TSS content (Table 1). In contrast, foliar treatments had significantly different impacts, and the least TSS content belonged to no-foliar treatment (*P* ≤ 0.05) (Table 3). Soluble solids act as osmolytes and cell structure protectors against oxidative stress factors. Chang et al^25^ demonstrated that trehalose application improved photosynthesis capability, transpiration, and stomatal conductance in *Catharanthus roseus*. Carbohydrates play a parental role in metabolic processes and gene expression, hence improving plant tolerance versus stressors^26^. Soluble solids can nourish metabolic pathways by producing NADPH and motivating the pentose-phosphate oxidative pathway, which scavenges and controls ROS radicals’ levels^27^. In strawberries, SA treatment improved TSS content of plants, consistent with our finding^28^. SA is involved in many physiological processes like controlling the absorption of several ions, stomata conductance, and membrane integrity and even has roles in photosynthesis potential and influences the biosynthesis of the secondary metabolites and TSS content^28,29^. Apart from being an energy source, glucose is a significant signaling molecule with various regulatory actions in the growth, development, and metabolic pathway in plants^29–32^.

**Table 3:** Mean comparisons for the effects of 2 g L^−1^ glucose, 2 g L^−1^ KNO_3,_ and 2% Dobogen foliar application on TSS content of *Tanacetum balsamita* plants grown hydroponically in perlite.

Foliar application	TSS (^0^Brix)
No-foliar spray	2.4^b^
Glucose	3.2^a^
KNO_3_	2.8^a^
Dobogen	2.7^a^

Significant differences among salinity treatments are indicated by the different Latin letters according to Duncan’s multiple range test.

### Chlorophyll’s content

**Figure 1:**
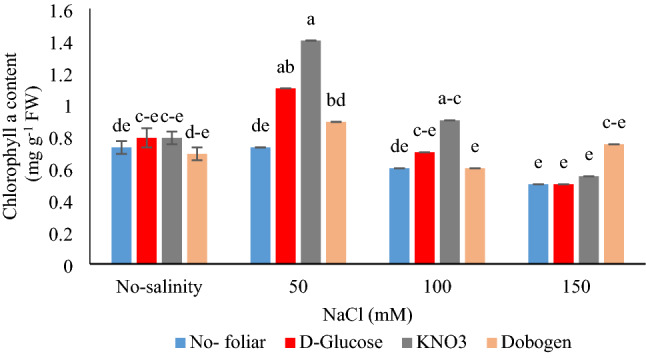
Interaction effect of salinity (0, 50, 100, and 150 mM NaCl) and foliar applications (no-foliar, 2 g L^−1^ glucose, 2 g L^−1^ KNO_3,_ and 2% Dobogen biostimulant) on the chlorophyll a content of *Tanacetum balsamita* plants grown in perlite. Significant differences among treatments are indicated by the different Latin letters according to Duncan’s multiple range test.

Figure 1 shows that the highest chl a content was recorded with 50 mM NaCl × foliar application of glucose and KNO_3_ and 100 mM NaCl × KNO_3_ treatment (*P* ≤ 0.01). Chl b was also affected by salinity (*P* ≤ 0.05) (Table 4). Up to 100 mM NaCl, there was no difference in chl b content (Table 5). Our results align with Aslam et al^15^ on sunflowers that salinity stress reduced photosynthesis and gas exchange in plants. The foliar use of KNO_3_ under saline-sodic conditions improved stomatal conductance, transpiration rate, water-use efficiency, CO_2_ fixation, and proline content^15^. The evaluation of the effect of the foliar treatment in the present study showed that foliar Dobogen treated plants and controls had the same data for chlorophyll a content (except NaCl_50 mM_). Furthermore, there was no difference between no-salinity treatment and 150 mM salinity × KNO3 and foliar glucose use for chlorophyll a content. Under 100 mM salinity, foliar KNO_3_ application was superior to Dobogen for chlorophyll a content. With increasing photosynthetic pigment content and accumulation, photochemical energy and metabolic activities are enhanced, and hence, the growth and productivity of plants reasonably improve^30,33^. In research on coriander (*Coriandrum sativum*), foliar treatment with KNO_3_ under salinity stress improved photosynthesis potential, ionic equilibrium, relative water content, and proteins biosynthesis. The idea is that potassium treatment regulates cell turgor and polarity, xylem translocation, and nitrogen metabolism, hence ameliorating salinity side-effects^30^. Chlorophyll content in plants is an indicator of abiotic stressors tolerance^33^. Abdallah et al. ^16^ noted that rice seed pretreatment with trehalose increased the chlorophyll content under salinity conditions. The increase in Rubisco enzyme activity and the consequently enhanced chlorophyll biosynthesis are the major reasons for the improved photosynthesis potential in the plants treated with trehalose, which ultimately led to the improved yield and productivity of plants^34^. It seems that trehalose mediates the physiological responses in the plant by the activation of several enzyme or metabolic pathways.

**Table 4:** ANOVA for the effects of salinity (0, 50, 100, and 150 mM NaCl) and foliar applications (no-foliar, KNO_3_, glucose, and Dobogen) on the chlorophyll a and b, total phenolics, flavonoids, proline, H_2_O_2,_ and MDA content as well as on SOD and CAT activity of *Tanacetum balsamita* plants grown hydroponically in perlite.

Source of variation	df	Chlorophyll a content	Chlorophyll b content	H_2_O_2_ content	MDA content	Flavonoids content	Total phenolics content	SOD activity	CAT activity	Proline con0tent
		Mean square	Mean square	Mean square	Mean square	Mean square	Mean square	Mean square	Mean square	Mean square
Salinity (S)	3	4.50**	6.60*	1913**	1907**	0.718**	403.848**	1.037**	3.859**	36,732.528**
Foliar (F)	3	6.90**	0.41^ns^	135**	150**	0.006^ns^	45.105*	0.120^ns^	1.488**	664.343**
S × F	9	0.14*	0.14^ns^	14^ns^	14.2^ns^	0.348**	84.074**	0.041^ns^	0.061^ns^	101.203^ns^
Error		0.034	0.025	0.084	7.001	0.113	11.330	0.107	0.166	92.221

df, degree of freedom; SOD, Superoxide dismutase; CAT, Catalase; ns, non-significant.

*Significant difference at *P* ≤ 5%, and **Significant difference at *P* ≤ 1%, following two-way ANOVA.

**Table 5:** Mean comparisons for the effects of salinity (0, 50, 100, and 150 mM NaCl) on chlorophyll b content, proline, and MDA content as well as on SOD and CAT activity of *Tanacetum balsamita* plants grown hydroponically in perlite.

Salinity levels (mM)	Chlorophyll b (mg g^−1^ FW)	Proline content (µg g^−1^ FW)	MDA content (nmol g^−1^ FW)	CAT activity (µmol H_2_O_2_ mg protein^−1^ min^−1^)	SOD activity (Units mg protein^−1^)
0	0.78^a^	40.7^d^	58.8^b^	2.13^c^	3.9^a^
50	0.72^a^	105.4^c^	56.3^c^	2.74^b^	3.47^b^
100	0.69^a^	129.2^b^	58.9^b^	3.1^ab^	3.53^b^
150	0.49^b^	151.4^a^	82.1^a^	3.24^a^	3.43^b^

Significant differences among salinity treatments are indicated by the different Latin letters according to Duncan’s multiple range test.

Moreover, trehalose-6- phosphate is a critical molecule in improving photosynthesis potential. Trehalose-6-phosphate acts as a key regulatory precursor in sugar influx and metabolism. Furthermore, the compound plays a crucial function in the redox activation of ADP-glucose pyrophosphorylase, a key enzyme in starch biosynthesis, and improves the photosynthesis rate and yield of plants^35^.

### Total phenolics and flavonoids content

Both total phenolics and flavonoids contents were impacted by salinity × foliar application (*P* ≤ 0.01) (Table 4). The highest phenolics content was traced by 50 mM NaCl without foliar spray and KNO_3_ foliar application and 100 mM NaCl × foliar application of Dobogen and glucose. The least phenolics content was recorded for 150 mM NaCl × no-salinity or KNO_3_ or Dobogen and in the treatment of no-salinity × KNO_3_. Glucose application was more influential on phenolics content under all-salinity levels than no-glucose application treatment.

**Figure 2:**
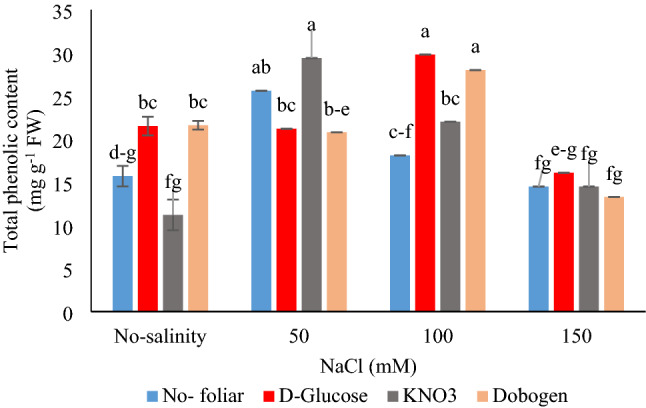
Interaction effects of salinity (0, 50, 100, and 150 mM NaCl) and foliar applications (no-foliar, 2 g L^−1^ glucose, 2 g L^−1^ KNO_3,_ and 2% Dobogen biostimulant) on the total phenolics content of *Tanacetum balsamita* plants grown in perlite. Significant differences among treatments are indicated by different Latin letters according to Duncan’s multiple range test.

**Figure 3:**
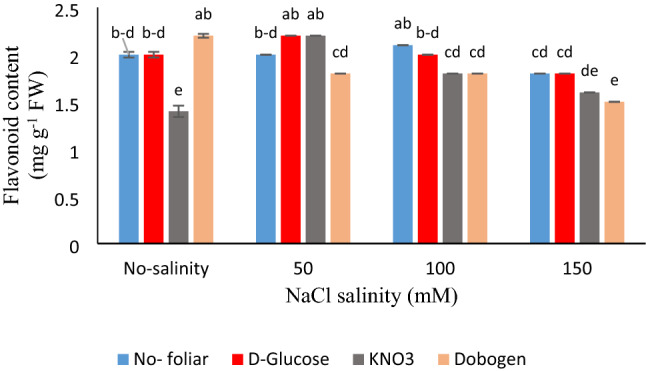
Interaction effects of salinity (0, 50, 100, and 150 mM NaCl) and foliar applications (no-foliar, 2 g L^−1^ glucose, 2 g L^−1^ KNO_3,_ and 2% Dobogen biostimulant) on flavonoids content of *Tanacetum balsamita* plants grown in perlite. Significant differences among treatments are indicated by different Latin letters according to Duncan’s multiple range test.

Moreover, considering phenolic content, foliar use of glucose and Dobogen acquired nearly the same results under all salinity levels (Fig. 2). The highest data for flavonoids were devoted to control plants’ foliar sprayed with Dobogen, 50 mM NaCl × KNO_3_ and glucose foliar treatment, and 100 mM salinity × no-foliar application (Fig. 3). The lowermost flavonoids content was recorded at no-salinity × KNO_3_ foliar application. In rosemary, with salinities of up to 50 mM, the total flavonoids content was increased^5^. Phenolics and flavonoids are the primary secondary metabolites that nullify oxidants, especially hydroxyl, peroxyl, and peroxynitrite radicals^23,36^. In *Catharanthus roseous*, the application of SA improved dry weight, water content, photosynthetic pigments, and proline content, as well as increased phenylalanine ammonia lyase (PAL) activity, which was coincident with phenolic biosynthesis stimulation in plant^2^. Furthermore, in *Solanum nigrum*, the expression of genes related to carotenoid and flavonoid biosynthesis (PAL, chalcone synthase, and flavonol synthase) was affected by salinity and further enhanced the accumulation of lutein and quercetine-3-β-D-glucoside. With 150 mM salinity, the amount of quercetin-3-β-D-glucoside was increased.

Meanwhile, lutein and β-carotene were negatively influenced by the mentioned salinity levels^23^, depicting the side effects of salinity on antioxidant compounds’ biosynthesis potential^23^. Salinity stress influences plants' physiological, biochemical, and cellular dynamics by imposing osmotic and ionic stresses and toxicity. Therefore, plants need to combat deteriorative salinity effects by modifying the genetic pathway and ion selection, distribution, and substitution and activating antioxidant systems^23,37^.

### Proline

Proline content was affected by the independent effects of salinity and foliar application (Table 4). The highest proline content was recorded at 150 mM NaCl, 26% higher than the control. For the foliar application treatments, KNO_3_ foliar spray was the most responsive (Table 6). There was no difference in proline content between the control and the treatments foliar sprayed with glucose and Dobogen. A convenient mechanism of stress tolerance in plants is the accumulation of compatible solutes like proline, which have prominent action in regulating osmotic balance in the cells, keeping the membranes integrity and enzyme/proline ratio, help in ROS scavenging, and assisting plants in protection versus stressors^30^. In coriander plant under salinity, proline content was increased^30^. Under salinity, the Na^+^ load considerably increases in the vacuoles. Therefore, the cells need parallel compounds with the same charge to control the osmotic potential of the cells. K^+^ availability inside cells induces proline biosynthesis by the related proteins hydrolysis^10,31^. Proline can scavenge free hydroxyl radicals and hence protects and stabilizes macromolecules such as DNA and proteins and secures cell membranes^32^.

**Table 6:** Mean comparisons for the effects of 2 g L^−1^ glucose, 2 g L^−1^ KNO_3,_ and 2% Dobogen foliar application on TSS, proline and MDA content, and catalase activity of *Tanacetum balsamita* plants grown hydroponically in perlite.

Foliar application	Proline content (µg g^−1^ FW)	CAT activity (µmol H_2_O_2_ mg protein^−1^ min^−1^)	MDA content (nmol g^−1^ FW)
No-foliar spray	100^b^	3.13^a^	69.1^a^
Glucose	104^b^	2.9^ab^	65.2^b^
KNO_3_	115^a^	2.7^bc^	59.7^d^
Dobogen	106^b^	2.41^c^	62.2^c^

Significant differences among treatments are indicated by different Latin letters.

### MDA content

MDA content was independently affected by salinity and foliar applications (Table 4). The salinity of 150 mM contained the highest MDA content (4% more than the control) (Table 5). Table 4 shows that the control plants with no-foliar spray treatments had the highest MDA content. Foliar treatments efficiently reduced MDA accumulation, and the lowest MDA amount belonged to the Dobogen foliar application, which was nearly 10% lower than the control (Table 6). In a study conducted on rosemary^5^ with increasing salinity, MDA accumulated more in the plant tissue. Under salinity conditions, ROS generation propagates via the enhanced enzymatic activity of membrane-anchored NADPH oxidases and peroxidases^27^. In costmary, salinity added up MDA production^21^. The simultaneous application of salinity and SA in *Ocimum basilicum* L. led to the reduced amounts of MDA, showing the ameliorative effects of SA on membrane integrity by the reduced genesis of ROS molecules^38^.

### SOD activity

SOD activity was influenced by the salinity treatments (Table 4). The highest data was recorded for control plants. There was no difference between salinity treatments considering SOD activity. With salinity increase up to 150 mM, SOD activity was reduced (12%) compared to the control, indicating the adverse effects of salinity on the enzyme activity and dynamics (Table 5). Plant survival under saline conditions depends on antioxidant enzyme activity, which scavenges ROS molecules. Salinity negatively influenced SOD activity in *Gossypium hirsutum*^39^*.* Foliar application of SA improved SOD activity in saline environments^42^. SOD is in the front line of struggling with the damages caused by ROS radicals and acts by converting O_2_^−^ into H_2_O_2_^43^. The produced H_2_O_2_ is then disassociated into H_2_O and O_2_ by the action of catalase. Otherwise, peroxidases neutralize H_2_O_2_ with the help and mediation of phenolics or other antioxidants^40^.

### CAT activity

Furthermore, CAT activity was responsive to the independent effects of salinity, and with salinities from 100 to 150 mM, the activity was increased. The lowest data for CAT was devoted to the control plants (Table 5). The highest CAT activity was recorded with the control plants and glucose foliar spray (Table 6). Our results are consistent with the reports on cucumber plants foliar treated with glucose under salinity. The foliar treatment improved catalase activity and seems to activate the stress-responsive genes^29^. As the final product of photosynthesis, glucose has a chief role in plants' growth regulation and physiological processes. Valizadeh Kamran et al.^21^ reported that salinity enhanced CAT activity in costmary. CAT is responsible for the catalysis of H_2_O_2_ with the help of ascorbate, guaiacol, and phenolics^41,47^.

### Na^+^, K^+^ content and K^+^/Na^+^ ratio

**Figure 4:**
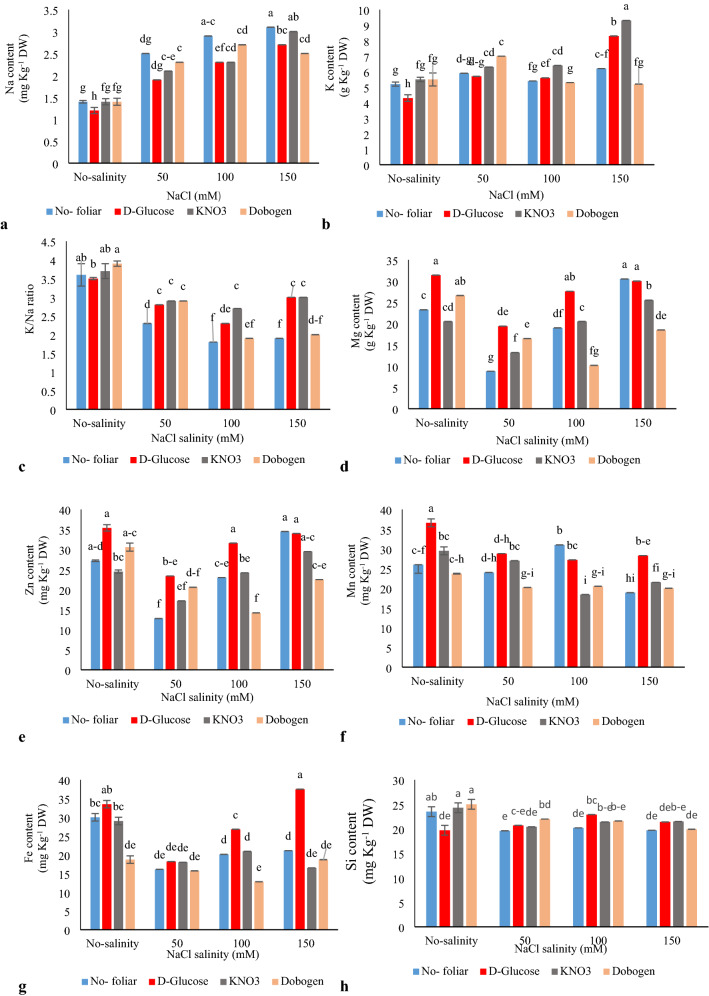
Interaction effect of salinity (0, 50, 100, and 150 mM NaCl) and foliar applications (no-foliar, 2 g L^−1^ glucose, 2 g L^−1^ KNO_3,_ and 2% Dobogen biostimulant) on the elemental content of *Tanacetum balsamita* plants grown in perlite. Significant differences among treatments are indicated by different Latin letters (*P* ≤ 0.01) according to Duncan’s multiple range test.

Na^+^ and K^+^ content and K^+^/Na^+^ ratio were influenced by salinity and foliar applications (Table 7). The highest K^+^ content was traced with 150 mM NaCl × KNO_3_ foliar application (77% more than control) (Fig. 4b). For Na^+^, 100 and 150 mM salinity × no-foliar spray and 150 mM NaCl × KNO_3_ were the statistically significant treatments (Fig. 4a). The highest K^+^/Na^+^ ratio belonged to the control (without salinity × without foliar spray) or without salinity × KNO_3_ and Dobogen foliar spray (Fig. 4c). In coriander, salinity influenced the contents of K, Mg, P, Ca, N, and Na/K ratio. KNO_3_ foliar application under the salinity stress improved N and K content of plants while reducing the Na/K ratio^30^. Similar results on the enhanced potassium levels under a saline environment and foliar SA spray have been reported in *Vigna unguiculata*^42^. Under saline-sodic conditions, Na^+^ enters the apoplastic lumens and, with substitution of Ca^2+^ ions in the cell membranes, depolarizes membranes and interferes with the selective absorption of essential minerals^43,44^. Salinity stress denatures and breakdowns membrane integrity and the excess K^+^ out-leakage stimulates polarization/activation of outward rectifying (KOR) K^+^ channels^43^. Keeping low Na^+^ and high K^+^ levels is the predominant goal-oriented criterion that mediate tolerance to salinity stress^37^. It seems that foliar treatment of plants with KNO_3_ is a feasible and reliable protocol to reduce the adverse effects of salinity via the enhanced K^+^/Na^+^ ratio.

**Table 7:** Effect of salinity (0, 50, 100, and 150 mM NaCl) and foliar applications (no-foliar, KNO_3_, glucose, and Dobogen) on the mineral content of *Tanacetum balsamita* plants grown hydroponically in perlite.

Source of variation	df	Na^+^	K^+^	K^+^/ Na^+^ ratio	Si	Mn^2+^	Zn^2+^	Fe^2+^	Mg^2+^	Ca^2+^
		Mean square	Mean square	Mean square	Mean square	Mean square	Mean square	Mean square	Mean square	Mean square
Salinity (S)	3	6.718**	13.023**	6.532**	21.797**	125.688**	330.667**	340.982**	0.007**	1.096^ ns^
Foliar (F)	3	0.535**	4.773**	1.343**	6.284*	226.949**	433.949**	429.511**	0.003**	3.082**
S × F	9	0.152**	4.118**	0.371**	9.369**	53.760**	100.005**	78.106*	0.001**	1.103**
Error		0.20	0.237	0.052	1.964	9.064	26.531	15.486	0.0001	0.706

df, degree of freedom; Na, Sodium; K, Potassium; Si, Silicon; Mn, Manganese; Zn, Zinc; Fe, Iron; Mg, Magnesium; Ca, Calcium; ns, non-significant.

*Significant difference at *P* ≤ 5% and **Significant difference at *P* ≤ 1%, following two-way ANOVA.

### Mg^2+^ content

Magnesium content was responded to the interaction of 150 mM salinity × no-foliar and glucose foliar application, 100 mM NaCl salinity × glucose spray, and no-salinity × Dobogen and glucose foliar use (Fig. 4d). Foliar treatments had no significant effects on Mg content of the plants. However, foliar spray at all salinity levels improved Mg content compared to no-foliar treatment. Elhindi et al.^30^ noted that foliar application of KNO_3_ improved Mg^2+^ content under salinity but reduced iron uptake and stimulated the dissociation of chlorophyll via photo-oxidation, blockage of chlorophyll biosynthesis, and over-activation of chlorophyll-catalyzing enzymes^18^.

### Mn^2+^ content

For Mn^2+^ content, the uppermost data belonged to foliar application with glucose × no-salinity stress (Fig. 4f). Salinity interferes with the intake of nutrients from the soil. Specifically, Na^+^ triggers strong osmotic effects and markedly decreases water and nutrient acquisition by impacting soil structure. Furthermore, Na^+^ replaces Ca^2+^ seating places in the cell structure and hence impedes the normal functions of cells and tissues^45–47^.

### Iron and silicon content

The iron content was increased by no-salinity × glucose and 150 mM NaCl × glucose interactions. It seems that foliar Dobogen application had the least effects on Fe content of plants compared to the other foliar used treatments (Fig. 4g). The top content for Si was recorded with no-salinity × no-foliar spray and with foliar spray of KNO_3_ and Dobogen (Fig. 4h). Several researches have verified that SA under the saline condition has crucial action in the physiological process, nutrients uptake, and even obstructs Na^+^ uptake and certifies plants survival under stressful environment^2^. With salinity, the Fe content of soybean plants drastically declined^45^. Iron plays crucial roles in plant growth, development, chlorophyll biosynthesis, thylakoid formation, and chloroplast development^30^. Overall, abiotic stressors impact plants by their effects on enzymatic, physiological, and biochemical activities and their influences on the antioxidant pool, photosynthesis, and ion homeostasis. Moreover, ionic imbalances caused by Cl^−^ and Na + over-accumulation hamper the absorption of other essential elements^5,8,45^. In aloe, Si application efficiently reduced the abortion and translocation of Na^**+**^ and Cl^−^ and simultaneously improved K^**+**^ content and K^**+**^/Na^−^ ratio. By the enhanced activation of protein pumps in the root cells, appropriate nutrition and especially suitable potassium supply protect cells and plants under stressful conditions. Furthermore, Si fortifies the cuticle layer in the sub-epidermal cells, delays or prevents water loss from the cells, protects cells against UV radiation, and ultimately assists plants in surviving under stress condition^46^.

### Zn^2+^ content

Zn^2+^ content was impacted by the interaction effects of no-saline × no-foliar treatments, no-saline × glucose and Dobogen, 150 mM salinity × no-foliar, and with glucose and KNO_3_ foliar spray as well as with 100 mM salinity × glucose foliar application (Fig. 4e). Zn^2+^ plays a pivotal role in membrane integrity and maintains a dominant leadership role in the regulated entrance of Na^+^ and other toxic ions into the cells. The appropriate Zn^2+^ availability is crucial for the survival of plants under saline-stressful environments. Since, with the optimized Zn^2+^ availability, the activity of NADPH, an enzyme responsible for the generation of some ROS types, greatly declines^48^. Salinity lessens Zn^2+^ absorption and concurrently diminishes the photosynthetic potential, stomatal conductance, respiration rate, chlorophyll content, and hormonal balance in plants^5,30^. The salinity × SA treatment in tomatoes showed that salinity reduced Zn, Fe, Ca, and K but increased Na content^19,49^. However, SA foliar application enhanced the content of Ca, K, Fe, and Zn. SA fulfills the action by maintaining the cell membrane's intactness under stressful environments. Furthermore, by mediating several physiological processes, SA prevents Na absorption and translocation in the main part by the activity enhancement of H^+^-ATP_ase_ in the root cells and simultaneously retains K efflux from the cells and, therefore, supports the plants withstand stressful environments^49^.

### Ca^2+^ content

**Figure 5:**
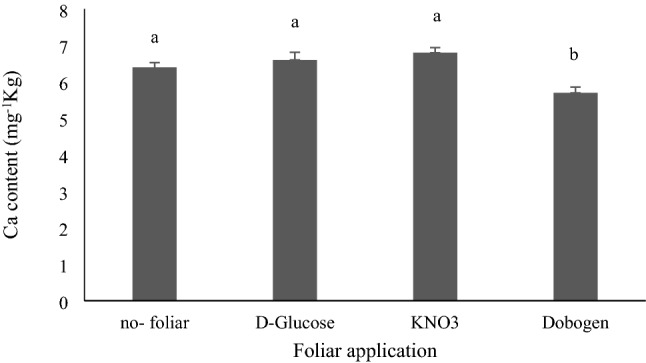
Mean comparisons for the effects of 2 g L^−1^ glucose, 2 g L^−1^ KNO_3_ and 2% Dobogen biostimulant foliar application on the calcium content of *Tanacetum balsamita* plants grown hydroponically in perlite. Significant differences among treatments are indicated by different Latin letters (*P* ≤ 0.01) according to Duncan’s multiple range test.

Salinity does not influence Ca^2+^ content. The highest Ca^2+^ content was recorded in no-foliar and foliar sprays with glucose and KNO_3_ (Fig. 5). Weisany et al.^45^ noted that Ca^2+^ absorption and accumulation declined with increasing salinity exposure in soybean roots. Aerial parts of plants are more sensitive to the unbalanced distribution of nutrients than the root system. The discrepancy in salt sensitivity and/or tolerance is species-dependent. It is linked with genetic makeup and specific gene expression in a defined plant taxon under stressful environments. The availability of appropriate amounts of Ca^2+^ is vital for cell membrane integrity and potential osmotic adjustment under saline conditions. The excessive Na^+^ availability in the rhizosphere medium and the subsequent intake of Na^+^ ions substitute cell-wall bonded Ca^2+^ with fake Na^+^ ions, which persuades magnificent devastation on cells, tissue, and plant organs and, subsequently, hinders the growth potential and productivity^45^. Elevated Ca^2+^ content as a secondary cellular messenger regulates the expression of specific salinity-dependent genes in favor of disciplined osmotic regulation, water absorption, ionic balance, and hence more acclimation to harsh saline environments in the main part by the appropriate responses of antioxidants, which improve Ca^2+^ intake for the maintenance of cell membranes integrity and viability against oxidative damages^39,50^.

### Essential oil yield and constituents

Essential oil yield was not affected by salinity, foliar application, or salinity × foliar application (Table 8). Salinity at 0 to 150 mM NaCl did not affect EO yield (averaged at 1.40 mL m^−2^) for plants grown under saline conditions (Table 8). Similarly, foliar application of glucose (averaged at 1.43 mL m^−2^) or Dobogen (averaged at 1.36 mL m^−2^) did not change the EO yield. In contrast, KNO_3_ application decreased the EO yield at a salinity of 50 mM NaCl compared with the relevant control (0 mM NaCl + KNO_3_).

The effects of different salinity levels and foliar application on the chemical composition of the EO of costmary are given in Table 9. The EO analysis identified 39 components for salinity, 40 components for glucose and KNO3 application, and 41 components for Dobogen application, showing that treatments represented 97.64–99.36% of the oils (Table 9). Oxygenated (monoterpenes and sesquiterpenes) compounds ranged from 89.38 to 94.50% and 0.82 to 3.11%, respectively, while hydrocarbon (monoterpenes and sesquiterpenes) compounds ranged from 0.53 to 1.71% and 1.13 to 3.44%, respectively (Table 9). The major constituents of the examined costmary EOs in decreasing order were carvone, cis-thujone, eucalyptol, trans-thujone, n-dodecane, tetradecane, trans-carvone oxide, and β-bisabolene. At the same time, the rest of the compounds were identified in amounts lower than 1% of the total volatile components (Table 9). Following statistical analysis, salinity affected the content of eucalyptol, trans-carvone oxide, and β-bisabolene. In contrast, neither the foliar application nor the interaction of salinity × foliar treatment affected the EO composition (Table 8).

**Table 8:** Effect of salinity levels (0, 50, 100 and, 150 mM NaCl) and foliar applications (no-foliar, 2 g L^−1^ glucose, 2 g L^−1^ KNO_3_, and 2% Dobogen) on the essential oil yield (mL/m^2^) and components (with > 1% content) in costmary grown in hydroponics.

Factors	Salinity (S)	Foliar (F)	Interaction S × F
EO yield	ns	ns	ns
carvone	ns	ns	ns
cis-Thujone	ns	ns	ns
Eucalyptol	*	ns	ns
trans-Thujone	ns	ns	ns
n-Dodecane	ns	ns	ns
Tetradecane	ns	ns	ns
trans-Carvone oxide	**	ns	ns
β-Βisabolene	**	ns	ns

ns, *, **, and *** indicate nonsignificant or significant differences at *P* < 5%, 1% and 0.1%, respectively, following two-way ANOVA.

Foliar Dobogen application in no-saline-grown plants increased the eucalyptol content compared with that in the no-sprayed plants. Similarly, KNO_3_ foliar application in no-saline-grown plants increased the trans-carvone oxide content compared to the control treatment (no-foliar) (Table 9). As the major secondary metabolites of essential oil-bearing plants, terpenoids respond to agricultural practices and environmental stimuli. Plants react to divergent environmental conditions by variations in chemical constituents' quantity and proportional ratio. Under varying agrarian practices, the fluctuations in the phytochemical profile of plants are quite logical as well. The genetic structure and phytochemical potential of plants inevitably mediate the chemical profile, albeit in coordination with environmental cues. Therefore, these sensing and signaling events lead to different physiological, biochemical, and yield-related responses. These fluctuations in the chemical profiles verify the enhanced adaptation process under stressful conditions. Therefore, the proportional variations in the oil constituents in response to the studied treatments are entirely acceptable.

**Table 9:** Chemical composition (%) of essential oils of costmary plants exposed to salinity (0, 50, 100, and 150 mM NaCl) and foliar applications (no-foliar, 2 g L^−1^ glucose, 2 g L^−1^ KNO_3_, and 2% Dobogen). Values (n = 3) in rows for each treatment followed by the same letter are not significantly different, *P* ≤ 0.05.

		No-foliar				D-Glucose				KNO_3_				Dobogen			
		Control	50 Mm	100 mM	150 mM	Control	50 mM	100 mM	150 mM	Control	50 mM	100 mM	150 mM	Control	50 mM	100 mM	150 mM
**Compound**	**Retention** **index**	**1**	**2**	**3**	**4**	**5**	**6**	**7**	**8**	**9**	**10**	**11**	**12**	**13**	14	**15**	**16**
Camphene	948	–	–	–	–	–	–	–	–	–	–	–	–	0.03a	0.00a	0.00a	0.00a
Sabinene	973	–	–	–	–	–	–	–	–	–	–	–	–	0.03a	0.00a	0.00a	0.00a
n-Decane	997	0.96ab	0.79ab	0.93ab	0.78ab	0.67ab	0.47ab	1.13ab	0.90ab	0.53ab	0.40b	0.78ab	1.12ab	1.19a	0.65ab	0.84ab	0.86ab
p-Cymene	1006	0.21ab	0.23ab	0.25ab	0.29ab	0.26ab	0.29ab	0.38a	0.30ab	0.33ab	0.13b	0.28ab	0.29ab	0.36a	0.33ab	0.30ab	0.32ab
Limonene	1028	0.05b	0.05b	0.06ab	0.10ab	0.08ab	0.09ab	0.19a	0.09ab	0.09ab	0.00b	0.05b	0.09ab	0.10ab	0.11ab	0.05b	0.05b
**Eucalyptol**	**1031**	**2.32bc**	**2.58bc**	**2.51bc**	**2.95ab**	**2.61bc**	**3.18ab**	**2.89ab**	**2.68abc**	**3.28ab**	**1.72c**	**2.44bc**	**2.58bc**	**3.66a**	**3.06ab**	**2.72abc**	**2.99ab**
Butanoic acid, 2-methyl-, 3-methylbutyl ester	1098	0.09abc	0.10abc	0.08abc	0.13a	0.04c	0.11abc	0.14a	0.13a	0.11abc	0.06bc	0.10abc	0.12ab	0.10abc	0.12ab	0.14a	0.10abc
Butanoic acid, 2-methyl-, 2-methylbutyl ester	1102	0.17de	0.18de	0.17de	0.26ab	0.17e	0.22abcde	0.26ab	0.21abcde	0.22abcde	0.25abc	0.20bcde	0.23abcd	0.19cde	0.21abcde	0.27a	0.21abcde
**cis-Thujone**	**1106**	**20.89a**	**20.09a**	**18.66a**	**20.69a**	**20.71a**	**18.50a**	**21.15a**	**20.74a**	**18.68a**	**18.60a**	**17.40a**	**18.68a**	**17.12a**	**20.95a**	**20.42a**	**20.08a**
**trans-Thujone**	**1116**	**2.20a**	**2.13a**	**2.07a**	**2.39a**	**2.25a**	**2.08a**	**2.45a**	**2.35a**	**2.15a**	**2.15a**	**2.01a**	**2.17a**	**2.06a**	**2.35a**	**2.41a**	**2.29a**
trans-p-Mentha 2,8-dien-1-ol	1119	0.31b	0.34ab	0.48ab	0.41ab	0.40ab	0.61a	0.53ab	0.53ab	0.57ab	0.45ab	0.60a	0.52ab	0.47ab	0.52ab	0.42ab	0.56ab
cis-p-Mentha -2,8-dien-1-ol	1133	0.11b	0.14ab	0.20ab	0.17ab	0.16ab	0.27a	0.23ab	0.23ab	0.25ab	0.19ab	0.27a	0.23ab	0.21ab	0.22ab	0.18ab	0.25ab
trans-Pinocarveol	1139	0.08c	0.18abc	0.22abc	0.26ab	0.22abc	0.29a	0.25ab	0.25ab	0.29a	0.11bc	0.26ab	0.23abc	0.26ab	0.25ab	0.22abc	0.26ab
Sabina ketone	1159	0.02bc	0.06abc	0.07abc	0.08abc	0.09abc	0.15a	0.12abc	0.14a	0.14a	0.00c	0.08abc	0.06abc	0.10abc	0.14ab	0.06abc	0.14a
Pinocarvone	1163	0.37c	0.42bc	0.50abc	0.58ab	0.51abc	0.53abc	0.57ab	0.50abc	0.55ab	0.49abc	0.47abc	0.56ab	0.59a	0.49abc	0.58ab	0.57ab
Thujol (3-thujanol)	1165	0.00b	0.06ab	0.05ab	0.15a	0.06ab	0.06ab	0.12ab	0.06ab	0.16a	0.00b	0.00b	0.00b	0.09ab	0.07ab	0.10ab	0.13ab
cis-Pinocarveol	1186	0.00c	0.00c	0.17bc	0.68ab	0.45abc	0.52abc	0.51abc	0.21bc	0.78a	0.35abc	0.38abc	0.42abc	0.61ab	0.36abc	0.33abc	0.41abc
a-Terpineol	1191	0.05ab	0.11ab	0.18a	0.12ab	0.18a	0.00b	0.12ab	0.00b	0.10ab	0.00b	0.00b	0.06ab	0.00b	0.09ab	0.00b	0.05ab
cis-Dihydro carvone	1198	0.57d	0.59 cd	0.62bcd	0.67abcd	0.71ab	0.66abcd	0.66abcd	0.66abcd	0.73a	0.62bcd	0.62bcd	0.66abcd	0.69abc	0.64abcd	0.67abcd	0.68abcd
**n-Dodecane**	1200	**1.57a**	**0.83a**	**1.38a**	**1.15a**	**0.72a**	**0.42a**	**1.06a**	**0.91a**	**0.50a**	**0.50a**	**1.01a**	**1.24a**	**1.14a**	**0.59a**	**0.94a**	**0.82a**
Verbenone	1211	0.00a	0.00a	0.00a	0.04a	0.04a	0.12a	0.07a	0.09a	0.05a	0.00a	0.00a	0.00a	0.00a	0.08a	0.04a	0.08a
trans-Carveol	1219	0.19c	0.19c	0.34abc	0.41abc	0.27bc	0.60ab	0.41abc	0.46abc	0.53abc	0.57abc	0.70a	0.43abc	0.38abc	0.37abc	0.22bc	0.45abc
cis-Carveol	1231	0.06c	0.09bc	0.23abc	0.23abc	0.17abc	0.30a	0.29a	0.25abc	0.29a	0.33a	0.33a	0.26ab	0.29a	0.20abc	0.22abc	0.30a
cis-Ocimenone	1232	0.09ef	0.09f.	0.12cdef	0.16bcdef	0.11def	0.22ab	0.19abcde	0.20abcd	0.19abcd	0.23ab	0.26a	0.16bcdef	0.17bcdef	0.16bcdef	0.16bcdef	0.20abc
Cumin aldeyde	1241	0.09ab	0.05bc	0.14a	0.13ab	0.12ab	0.00c	0.00c	0.00c	0.07abc	0.00c	0.00c	0.00c	–	–	–	–
**Carvone**	**1244**	**63.33a**	**65.52a**	**64.47a**	**61.56a**	**63.32a**	**64.32a**	**60.15a**	**60.80a**	**63.90a**	**61.68a**	**62.36a**	**62.10a**	**63.32a**	**62.02a**	**60.71a**	**62.27a**
cis-Chrysanthenyl acetate	1259	0.07bc	0.06c	0.10abc	0.13abc	0.13abc	0.13abc	0.18a	0.15a	0.12abc	0.16a	0.16a	0.18a	0.13abc	0.15a	0.16a	0.14ab
cis-Carvone oxide	1262	0.00d	0.03 cd	0.00d	0.04bcd	0.09abc	0.10abc	0.06abcd	0.11abc	0.11abc	0.00d	0.05abcd	0.10abc	0.10bc	0.12ab	0.11ab	0.13a
Perilla aldehyde	1275	–	–	–	–	0.03ab	0.00b	0.00b	0.00b	0.03ab	0.00b	0.00b	0.00b	0.07a	0.00b	0.00b	0.04ab
**trans-Carvone oxide**	**1276**	**0.93b**	**1.10ab**	**1.08ab**	**1.07ab**	**1.25ab**	**1.43a**	**1.23ab**	**1.31a**	**1.31a**	**1.36a**	**1.31a**	**1.15ab**	**1.23ab**	**1.33a**	**1.17ab**	**1.34a**
trans-Carvyl acetate	1335	0.00a	0.00a	0.04a	0.04a	0.06a	0.05a	0.05a	0.00a	0.05a	0.00a	0.07a	0.10a	0.06a	0.06a	0.06a	0.04a
cis-Carvyl acetate	1360	0.02d	0.05 cd	0.14abcd	0.13abcd	0.16abc	0.14abcd	0.18ab	0.08bcd	0.17abc	0.24a	0.20ab	0.17abc	0.13abcd	0.12bcd	0.15abc	0.15abc
**Tetradecane**	1397	**1.14a**	**0.47a**	**1.01a**	**0.70a**	**0.38a**	**0.19a**	**0.55a**	**0.52a**	**0.20a**	**0.37a**	**0.70a**	**0.76a**	**0.56a**	**0.25a**	**0.55a**	**0.43a**
**β-Βisabolene**	**1520**	**1.38bc**	**1.38bc**	**1.27bc**	**1.19bc**	**1.06bc**	**0.95c**	**1.08bc**	**1.48bc**	**1.56bc**	**2.98a**	**2.21abc**	**2.33ab**	**1.97abc**	**1.76abc**	**2.10abc**	**1.37bc**
trans-Calamenene	1531	0.22bc	0.21bc	0.18bc	0.21bc	0.18c	0.18c	0.24abc	0.26abc	0.25abc	0.46a	0.43ab	0.40abc	0.37abc	0.30abc	0.36abc	0.21bc
Spathulenol	1581	0.11bcd	0.08 cd	0.12bcd	0.16bcd	0.18bcd	0.19bcd	0.22bcd	0.31bcd	0.16bcd	0.62a	0.36b	0.24bcd	0.13bcd	0.05d	0.32bc	0.17bcd
Caryphylllene oxide	1587	0.00b	0.01b	0.03b	0.00b	0.02b	0.11ab	0.00b	0.13ab	0.05ab	0.17a	0.07ab	0.06ab	0.06ab	0.03b	0.08ab	0.00b
Hexadecane	1597	0.37a	0.16a	0.32a	0.18a	0.12a	0.00a	0.17a	0.17a	0.00a	0.15a	0.24a	0.24a	0.18a	0.06a	0.19a	0.13a
1 epi-Cubenol	1628	0.08 cd	0.06 cd	0.04 cd	0.09 cd	0.08 cd	0.06 cd	0.07 cd	0.17abcd	0.00d	0.30a	0.25ab	0.16abcd	0.07 cd	0.12bcd	0.18abc	0.06 cd
β-Cedren-9-one	1634	0.00b	0.00b	0.00b	0.05b	0.00b	0.00b	0.00b	0.12ab	0.00b	0.22a	0.11ab	0.00b	0.00b	0.00b	0.12ab	0.00b
a epi-cadinol	1638	0.42abc	0.39abc	0.35abc	0.35abc	0.22abc	0.15bc	0.36abc	0.43abc	0.15bc	0.65a	0.52ab	0.36abc	0.00c	0.00c	0.29abc	0.00c
a Cadinol	1657	0.65a	0.54a	0.57a	0.64a	0.66a	0.63a	0.71a	0.90a	0.57a	1.15a	1.12a	0.68a	0.69a	0.63a	0.87a	0.60a
Total Identified		99.13	99.38	99.17	99.36	98.96	98.31	98.97	98.80	99.20	97.64	98.40	99.14	98.90	98.96	98.69	98.88
Monoterpene hydrocarbons		1.22abc	1.06abc	1.26abc	1.17abc	1.01abc	0.84bc	1.70a	1.29abc	0.95abc	0.53c	1.12abc	1.50ab	1.71a	1.08abc	1.18abc	1.24abc
Oxygenated monoterpenes		91.87ab	94.05a	92.42ab	93.22ab	94.03a	94.25a	92.48ab	91.82ab	94.50a	89.38b	89.97ab	90.82ab	91.74ab	93.73ab	91.22ab	93.55ab
Sesquiterpenes hydrocarbons		1.59bc	1.59bc	1.46bc	1.40bc	1.24bc	1.13c	1.32bc	1.75bc	1.81bc	3.44a	2.63abc	2.74ab	2.34abc	2.06abc	2.46abc	1.58bc
Oxygenated sesquiterpenes		1.27bc	1.09bc	1.12bc	1.29bc	1.17bc	1.14bc	1.36bc	2.05abc	0.93c	3.11a	2.42ab	1.50bc	0.95c	0.84c	1.86abc	0.82c
Others		3.27a	1.64a	3.06a	2.41a	1.63a	0.94a	2.12a	1.90a	1.07a	1.18a	2.26a	2.58a	2.16a	1.24a	1.97a	1.69a

Significant values are in [bold].

Overall, salinity stress impressed root dry weight, chlorophyll b content, proline content, catalase activity, malondialdehyde content, leaf length, plant height, and superoxide dismutase activity. Moreover, salinity and foliar application interactions affected the chlorophyll a, total phenolics and flavonoid content, sodium and potassium contents, potassium to sodium ratio, magnesium content, and zinc, iron, and manganese content. Although salinity did not affect the dry weight of the aerial part of the plant, it significantly affected most of the mentioned physiological traits. In some foliar spraying treatments, salinity increased the predominant components of the essential oil, such as eucalyptol. In contrast, with other treatments, the major components declined, indicating that the essential oil constituents' variations in response to salinity stress and foliar applications were treatment dependent.

## Material and methods

This experiment was conducted during the spring and summer of 2019 at the Research Greenhouse of Azarbaijan Shahid Madani University, Tabriz, Iran. The greenhouse growing conditions were as follows: lightening period: 16:8 day and night, temperature regime, 30 °C and 25 °C day and night, and relative humidity of approximately 65 ± 5%.

### Plant material and experimental setup

The homogeneous costmary (*Tanacetum balsamita* L.) rhizomes were provided by a commercial cultivation site in Azar-shahr County, Northwest Iran, according to the relevant institutional and national guidelines and legislation. The rhizomes were divided into uniform sections (approximately 10 cm in size) and surface sterilized with sodium hypochloride (NaCl; 5% v/v) for 20 min followed by washing with distilled water. Rhizomes were planted in 5-L pots containing medium-sized perlite. During the early establishing growth stage, the plants were nourished with half-strength Hoagland’s nutrient solution. Afterward, following a period of 3 weeks, salinity treatments were imposed. The salinity levels were 0, 50, 100, and 150 mM NaCl. Salinity began at 25 mM and gradually increased to reach the final level within 10 days. Salinity levels were based on the high salinity damage record in Northwest Iran and previous studies^5^. The pots were regularly washed with tap water once every week to avoid salinity standup on the pot surfaces. Fertigation was applied during the daytime adapted according to the plant needs and growth stage.

The optimal pH of the nutrient solution (NS) was 5.8 and was recorded every day and adjusted accordingly by using H_2_SO_4_ (5% v/v). Following salinity application, the electrical conductivity (EC) of NS was 2.1 mS cm^−1^ (0 mM NaCl), 6.0 mS cm^−1^ (50 mM NaCl), 12.0 mS cm^−1^ (100 mM NaCl), and 19.0 mS cm^−1^ (150 mM NaCl). Foliar treatments were applied as sprays, and the solutions used included; dH_2_O, 2 g L^−1^ KNO_3_, 2 g L^−1^ glucose, and 2% Dobogen biostimulant (Arman Sabz Adineh, Iran). Concentration levels were chosen based on preliminary experiments and previous records (unpublished data from our lab). Dobogen contains 10% salicylic acid, 0.05% soluble boron and 0.005% soluble molybdenum. Foliar applications were applied twice during the plant growth period. The first application was just after salinity exposure, and the second foliar spray was three weeks later. The plant samples were taken for further analysis one month after the second foliar treatment (at seven weeks of salt stress). Every pot contained a single rhizome of 10 cm in length × approximately 1 cm in diameter. Each experimental unit consisted of two pots, and the treatments had three replications.

### The fresh and dry weight of plants (biomass)

The plants were harvested at the early flowering stage. The aboveground and belowground parts of the plants were separated, weighed, and air-dried until reaching a constant weight. The fresh and dry biomass of plant organs was recorded by a digital scale (BB141, Boeco, Germany). Furthermore, plant height, root dry weight (oven-dried until constant weight), petiole length, and leaves' length and width were recorded at the harvest time.

### Chlorophyll content

Chlorophylls a and b were quantified by the method of Prochazkova et al.^51^ by a spectrophotometer (T80^+^, Beijing, China) at 645 (chlorophyll b) and 665 (chlorophyll a) nm. Leaf samples (0.5 g) were extracted by dimethyl sulfoxide (DMSO, Sigma Aldrich, Germany) in the dark for 4 h at 65 °C, and the results were expressed in mg per g of fresh weight (mgg^−1^ FW).

### Soluble solids content of the leaves

Soluble solids content (TSS) was quantified by a hand refractometer (Erma, Tokyo, Japan) from the extract obtained by squeezing the leaves, and the data are presented as ^0^Brix.

### Elemental composition

Leaves were dried at 75 °C for 4 d, weighed, and ground in a Wiley mill to particles less than 0.42 mm. Subsamples (0.2–0.3 g) were acid digested (2 N HCl) and analyzed for nutrient content as described in Chrysargyris et al.^52^. The contents of sodium (Na) and potassium (K) were quantified by the flame photometric method (Corning, 410, England). The contents of magnesium (Mg), calcium (Ca), and iron (Fe) were measured by atomic absorption spectroscopy (Shimadzu, AA6300, Tokyo, Japan) as previously described by Honarjoo et al.^53^, phosphorus (P) by vanadate molybdate^53^, and nitrogen (N) content by the Kjeldahl method^53^.

### Hydrogen peroxide and lipid peroxidation

The content of hydrogen peroxide (H_2_O_2_) was assessed according to Arjunan et al.^54^. Leaf tissue (0.2 g) was powdered in liquid N_2_, ground in ice-cold 0.1% trichloroacetic acid (TCA), and centrifuged at 12,000 g for 15 min. Aliquots (0.5 mL) of the supernatant were mixed with 0.5 mL of 10 mM potassium phosphate buffer (pH = 7.5) and 1 mL of 1 M potassium iodide. The H_2_O_2_ concentration was evaluated using standards of 5 to 1000 μM H_2_O_2,_ and a calibration curve was plotted accordingly. The absorbance of the samples and standards was measured at 390 nm, and the results were expressed as μmol H_2_O_2_ g^−1^ fresh weight.

Lipid peroxidation was determined as described by Azevedo-Neto et al.^55^ in terms of malondialdehyde content (MDA). Leaf tissue (0.2 g) was homogenized in 0.1% TCA, and the extract was centrifuged at 12,000 g for 15 min. The reaction mixture of 0.5 mL extract and 1.5 mL of 0.5% thiobarbituric acid (TBA) in 20% TCA was incubated at 95 °C for 30 min and then cooled in an ice bath. The absorbance was determined at 520 nm and corrected for nonspecific absorbance at 600 nm. The MDA amount was determined using an extinction coefficient of 155 mM cm^−1^. The results were expressed as nmol of MDA/g fresh weight.

### Superoxide dismutase (SOD) and catalase (CAT) activity

SOD activity was traced by recording the inhibition of nitroblue tetrazolium (NBT) photoreduction by the enzyme. The reaction mixture contained 50 mM sodium phosphate buffer (pH 7.6), 0.1 mM EDTA, 50 mM sodium carbonate, 12 mM L-methionine, 50 µM NBT, 10 µM riboflavin and 100 µL of plant sample extract in a final volume of 3.0 mL. SOD activity was recorded at 560 nm by a spectrophotometer. One unit (U) of SOD activity was defined as the amount of enzyme causing 50% inhibition of photochemical reduction of NBT^56^. CAT activity was recorded by monitoring the absorbance decline at 240 nm due to H_2_O_2_ scavenging. The activity was expressed as units (U) of catalase, which caused an absorbance change of 0.001 per min. The reaction mixture contained 100 mM sodium phosphate buffer (pH 7.0), 30 mM H_2_O_2,_ and 100 µL of the plant extract with a final volume of 3.0 mL^23^.

### Total phenolics and flavonoids content

A methanolic extract of plant tissue (0.5 g) was used to quantify the phenolic content by Folin-Ciocalteu reagent at 755 nm, according to Kim et al.^57^. The results were expressed as equivalents of gallic acid (Scharlau, Barcelona, Spain) per g of plant dry weight (mg of GAE/g dry weight).

Total flavonoids were assayed according to the aluminum chloride colorimetric method^57^, and the absorbance was recorded at 510 nm. The content of total flavonoids is expressed as rutin equivalents (mg rutin/g dry tissue).

### Proline content

The proline content was assayed according to acid-ninhydrin and toluene at 520 nm, as described by Fedina et al.^58^. The proline content was computed using a standard curve of proline, and the results were expressed as micrograms of proline per gram of plant fresh weight.

### Essential oil extraction and analysis

Air-dried plant samples (50 g) were hydro-distilled by a Clevenger-type apparatus from the European pharmacopoeia for 3 h. The oils were dried over anhydrous sodium sulfate and kept in sealed airtight amber vials until analysis. The EO yield was measured (mL m^−2^), oils were analyzed by gas chromatography-mass spectrometry (GC/MS- Shimadzu GC2010 gas chromatograph interfaced with Shimadzu GC/MS QP2010 plus mass spectrometer), and the constituents were determined as described previously^26^.

### Experimental design and data analysis

The experiment was factorial based on a completely randomized design with three replications, and each replication was a pool of two plant tissue samples as biological replications. Data were analyzed by MSTATC and SPSS (ver.15), and means were compared by Duncan's multiple range test at *P* ≤ 0.05 and *P* ≤ 0.01%. The graphs were drawn by Microsoft Excel, 2013.

## Conclusions

The overall results showed the ameliorative effects of foliar treatments on the salinity depression of costmary plants. Salinity profoundly influenced plant height, leaf length and width, proline content, CAT and SOD activity, and MDA and chlorophyll b content. Moreover, the treatments impacted total phenolics and flavonoids, chlorophyll a, Si, Fe, Mn, and Mg content, and K^+^/Na^+^ ratio. Na^+^ content was responsive to the interaction of salinity × foliar applications. Costmary was relatively tolerant to salinity depression, and foliar application of KNO_3_ and glucose would be cost-effective feasible alternatives to enhance salt tolerance and to improve the growth responses and productivity of costmary. Altogether, costmary was tolerant to the mild stress levels. Foliar treatments effectively mitigated the salinity side-effects under the low to mild salinity levels. Still, the foliar treatments with high salinity levels exposure were not potentiated to smoothen the adverse salinity effects. Considering, more detailed studies with a broad range of foliar treatments and even with novel compounds are needed to decide on the efficiency and extendibility of foliar treatments. Later, with the comprehensive, detailed studies, the result may be advisable to the extension section and pioneer farmers to secure the sustainable production of this plant under saline-prone soils and environments.

## Data Availability

All-new research data are presented in this contribution.

## References

[1] Hassanpouraghdam MB, Tabatabaei SJ, Nazemyieh H, VojodiMehrabani L, Azami A (2009). Volatile oil constituents of alecost [*Tanacetum balsamita* L. ssp. balsamitoides (Schultz-Bip.)] growing wild in North-West of Iran. Herba Pol..

[2] Mallahi T, Saharkhiz MJ, Javanmardi J (2018). Salicylic acid changes morpho-physiological attributes of feverfew (*Tanacetum parthenium* L.) under salinity stress. Acta Ecol. Sin..

[3] Khan MA, Ungar IA, Showalter AM (2000). The effect of salinity on the growth, water status and ion content of a leaf succulent perennial halophyte, *Suaeda fruticosa* L. Forsk. J. Arid Environ..

[4] Parida AK, Das AB (2005). Salt tolerance and salinity effects on plants: A review. Ecotoxicol. Environ. Saf..

[5] Vojodi Mehrabani L, Hassanpouraghdam MB, Shamsi-Khotab T (2018). The effects of common and nanozinc foliar application on the alleviation of salinity stress in *Rosmarinus officinalis* L. Acta Sci. Pol. Hortoru Cultus..

[6] Turkan I, Demiral T (2009). Recent developments in understanding salinity tolerance. Environ. Exp. Bot..

[7] Chen H, Jiang JG (2010). Osmotic adjustment and plant adaptation to environmental changes related to drought and salinity. Environ. Rev..

[8] Chrysargyris A, Michailidi E, Tzortzakis N (2018). Physiological and biochemical responses of *Lavandula angustifolia* to salinity under mineral foliar application. Front. Plant Sci..

[9] Chrysargyris A, Solomou M, Petropoulos S, Tzortzakis N (2019). Physiological and biochemical attributes of *Mentha spicata* when subjected to saline conditions and cation foliar application. J. Plant Physiol..

[10] Maathuis FJM, Amtmann A (1999). K^+^ nutrition and Na^+^ toxicity: The basis of cellular K^+^/Na^+^ ratios. Annal. Bot.

[11] Kaya C, Tuna AL, Ashraf M, Altunlu H (2007). Improved salt tolerance of melon (*Cucumis melo* L.) by the addition of proline and potassium nitrate. Environ. Exp. Bot..

[12] Hassanpouraghdam MB, Vojodi Mehrabani L, Tzortzakis N (2019). Foliar application of nano zinc and iron affects physiological attributes of *Rosmarinus officinalis* and quietens NaCl salinity depression. J. Soil Sci. Plant Nutr..

[13] Valizadeh Kamran R, Vojodi Mehrabani L, Pessarakli M (2019). Effects of foliar application of methanol on some physiological characteristics of *Lavandula stoechas* L. under NaCl salinity conditions. J. Plant Nutr..

[14] Chrysargyris A, Xylia P, Botsaris G, Tzortzakis N (2017). Antioxidant and antibacterial activities, mineral and essential oil composition of spearmint (*Mentha spicata* L.) affected by the potassium levels. Ind. Crops Prod..

[15] Aslam A, Khan Sh, Ibrar D, Irshad S, Bakhsh A, Gardezi STR, Ali M, Hasnain Z, Al-Hashimi A, Noor MA, Brestic M, Skalicky M, Zuan ATK (2021). Defensive impact of foliar applied potassium nitrate on growth linked with improved physiological and antioxidative activities in sunflower (*Helianthus annuus* L.) hybrids grown under salinity stress. Agronomy.

[16] Luo Y, Li F, Wang GP, Yang XH, Wang W (2010). Exogenously supplied trehalose protects thylakoid membranes of winter wheat from heat-induced damage. Biol. Plant..

[17] Abdallah MMS, Abdelgawad ZA, El-Bassiouny HMS (2016). Alleviation of the adverse effects of salinity stress using trehalose in two rice varieties. S. Afr. J. Bot..

[18] Mascellani A, Natali L, Cavallini A, Mascagni F, Caruso G, Gucci R, Havlik J, Bernardi R (2021). Moderate salinity stress affects expression of main sugar metabolism and transport genes and soluble carbohydrate content in ripe fig fruits (*Ficus carica* L. cv. Dottato). Plants.

[19] Senaratna T, Touchell D, Bunn E, Dixon K (2000). Acetylsalicylic (aspirin) and salicylic acid induce multiple stress tolerance in bean and tomato plants. Plant Growth Regul..

[20] Neocleous D, Vasilakakis M (2007). Effects of NaCl stress on red raspberry (*Rubus idaeus* L. "Autumn Bliss"). Sci Hortic..

[21] Valizadeh Kamran R, Vojodi Mehrabani L, Hassanpouraghdam MB, Pessarakli M (2017). Effects of foliar application of FeSO_4_ and NaCl salinity on vegetative growth, antioxidant enzymes activity, and malondialdehyde content of *Tanacetum balsamita* L. Commun. Soil Sci. Plant Anal..

[22] Chrysargyris A, Papakyriakou E, Petropoulos SA, Tzortzakis N (2019). The combined and single effect of salinity and copper stress on growth and quality of *Mentha spicata* plants. J. Hazard. Mater..

[23] Ben Abdallah S, Aung B, Amyot L, Lalin L, Lachaal M, Karray-Bouraoui N, Hannoufa A (2016). Salt stress (NaCl) affects plant growth and branch pathways of carotenoid and flavonoid biosynthesis in *Solanum nigrum*. Acta Physiol. Plant.

[24] Vojodi Mehrabani L, Valizadeh Kamran R, Hassanpouraghdam MB, Pessarakli M (2017). Zinc sulphate foliar application effects on some physiological characteristics and phenolic and essential oil contents of *Lavandula stoechas* L. under sodium chloride (NaCl) salinity conditions. Commun. Soil Sci. Plant Anal..

[25] Chang B, Yang L, Cong W, Zu Y, Tang Z (2014). The improved resistance to high salinity induced by trehalose is associated with ionic regulation and osmotic adjustment in *Catharanthus roseus*. Plant Physiol. Biochem..

[26] Faisal S, Mahboob W, Ud-Din Khan Z (2018). Efficacy of exogenous application on Glucose in improving wheat crop (*Triticum aestivum* L.) performance under drought stress. Pak. J. Agric. Res..

[27] Durner J, Klessig DF (1996). Salicylic acid is a modulator of tobacco and mammalian catalases. Plant Mol. Biol..

[28] Metwally AA, Youssef SM, El-Miniawy SM, Ragab ME (2013). Effect of foliar spraying of salicylic acid on growth, yield, and quality of cold stored strawberry plants. J. Biol. Chem. Environ. Sci..

[29] Ma S, Guo S, Chen J, Sun J, Wang Y, Shu S (2020). Enhancement of salt-stressed cucumber tolerance by application of glucose for regulating antioxidant capacity and nitrogen metabolism. Can. J. Plant Sci..

[30] Elhindi KM, El-Hendawy S, Abdel-Salam E, Schmidhalter U, Rehman S, Adl Hassan A (2016). Foliar application of potassium nitrate affects the growth and photosynthesis in coriander (*Coriander sativum* L.) plants under salinity. Prog. Plant Nutr..

[31] Idrees M, Naeem M, Khan MN, Aftab T, Khan MMA, Moinuddin M (2012). Alleviation of salt stress in lemongrass by salicylic acid. Protoplasma.

[32] Misra N, Misra R, Mariam A, Yusuf K, Yusuf L (2014). Salicylic acid alters antioxidant and phenolics metabolism in *Catharanthus Roseus* grown under salinity stress. Afr. J. Tradit. Complement. Altern. Med..

[33] Shah SH, Hourborg R, McCabe MF (2017). Response of chlorophyll, carotenoid and SPAD-502 measurement to salinity and nutrient stress in wheat (*Triticum aestivum* L.). Agronomy.

[34] Paul M, Primavesi LF, Jhurreea D, Zhang Y (2008). Trehalose metabolism and signaling. Annu. Rev. Plant Biol..

[35] Grennan A (2007). The role of trehalose biosynthesis in plant. Plant Physiol..

[36] Carocho M, Ferreira ICFR (2013). A review on antioxidants, prooxidants and related controversy: Natural and synthetic compounds, screening and analysis methodologies and future perspectives. Food Chem. Toxicol..

[37] Sun W, Xu X, Zhu H, Liu A, Liu L, Li J, Hua X (2010). Comparative transcriptomic profiling of salt-tolerant wild tomato species and salt-sensitive tomato cultivar. Plant Cell Physiol..

[38] Delavari Parizi M, Baghizadeh A, Enteshari S, Kalantari KM (2012). The study of the interactive effects of salicylic acid and salinity stress on induction of oxidative stress and mechanisms of tolerance in *Ocimum basilicum* L. J. Plant Biol..

[39] Dong YJ, Wang ZL, Zhong JW, Liu S, He ZL, He MR (2015). Interaction effects of nitric oxide and salicylic acid in alleviating salt stress of *Gossypium hirsutum* L. J. Soil Sci. Plant Nutr..

[40] Tarchoune I, Sgherri C, Izzo R, Lachaal M, Navari-Izzo F, Ouerghi Z (2012). Changes in the antioxidative systems of *Ocimum basilicum* L. (cv. Fine) under different sodium salts. Acta Physiol. Plant..

[41] Kang HM, Saltveit ME (2002). Chilling tolerance of maize, cucumber and rice seedling leaves and roots are differentially affected by salicylic acid. Physiol. Plant..

[42] El-Taher AM, El-Raouf HSA, Osman NA, Azoz SN, Omar MA, Elkelish A, El-Hady MAMA (2022). Effect of salt stress and foliar application of salicylic acid on morphological, biological, anatomical and productivity characteristics of cowpea (*Vigna unguiculata) plants*. Plants.

[43] Shabala S, Cuin TA (2008). Potassium transport and plant salt tolerance. Plant Physiol..

[44] Ashraf M, Foolad MR (2007). Role of glycine betaine and proline in improving plant abiotic stress resistance. Environ. Exp. Bot..

[45] Weisany W, Sohrabi Y, Heidari GR, Siosemardeh A, Badakhshan H (2014). Effects of zinc application on growth, absorption and distribution of mineral nutrients under salinity stress in soybean (*Glycine Max* L.). J. Plant Nutr..

[46] Xu CX, Ma YP, Liu YL (2015). Effects of silicon (Si) on growth, quality and ionic homeostasis of aloe under salt stress. S. Afr. J. Bot..

[47] Sairam RK, Srivastava GC, Agarwal S, Meena RC (2005). Difference in antioxidant activity in response to salinity stress in tolerant and susceptible wheat genotypes. Biol. Plant..

[48] Cakmak I, Marschner H (1988). Zinc-dependent changes in ESR signals, NADPH oxidase and plasma membrane permeability in cotton roots. Physiol. Plant..

[49] Souri MK, Tohidloo Gh (2019). Effectiveness of different methods of salicylic acid application on growth characteristics of tomato seedling under salinity. Chem. Biol. Technol. Agric..

[50] El-Tayeb M (2005). Response of barley grains to the interactive effect of salinity and salicylic acid. Plant Growth Regul..

[51] Prochazkova D, Sairam RK, Srivastava GC, Singh DV (2001). Oxidative stress and antioxidant activity as the basis of senescence in maize leaves. Plant Sci..

[52] Chrysargyris A, Panayiotou C, Tzortzakis N (2016). Nitrogen and phosphorus levels affected plant growth, essential oil composition, and antioxidant status of lavender plant (*Lavandula angustifolia* Mill.). Ind. Crops Prod..

[53] Honarjoo N, Hajrasuliha Sh, Amini H (2013). Three plants in absorption of ions from different natural saline and sodic soils. Int. J. Agric. Crop. Sci..

[54] Arjunan NK, Murugan K, Baruah I, Madhiyazhagan P, Thiyagarajan N (2012). Larvicidal potentiality, longevity and fecundity inhibitory activities of *Bacillus sphaericus* (Bs G3-IV) on vector mosquitoes, *Aedes aegypti* and *Culex quinque* fasciatus. J. Entomolo. Acarol. Res..

[55] Azevedo Neto AD, Prisco JT, Enéas-Filho J, Abreu CEBD, Gomes-Filho E (2006). Effect of salt stress on antioxidative enzymes and lipid peroxidation in leaves and roots of salt-tolerant and salt-sensitive maize genotypes. Environ. Exp. Bot..

[56] Alici EH, Arabaci G (2016). Determination of SOD, POD, PPO and CAT enzyme activities in *Rumex obtusifolius* L. Annu. Res. Rev. Biol..

[57] Kim KH, Tsao R, Yang R, Cui SW (2006). Phenolic acid profiles and antioxidant activities of wheat bran extracts and the effect of hydrolysis conditions. Food Chem..

[58] Fedina I, Georgieva K, Velitchkova M, Grigorova I (2006). Effect of pretreatment of barley seedlings with different salts on the level of UV-B induced and UV-B absorbing compounds. Environ. Exp. Bot..

